# The designing and modeling of equal base circle herringbone curved bevel gears

**DOI:** 10.1038/s41598-023-28934-0

**Published:** 2023-01-31

**Authors:** Ruiping Zhang, Bo Zhang, Shixiang Fu

**Affiliations:** School of Mechatronics Engineering, Henan University of Science and Technology, Luoyang, 471000 China

**Keywords:** Mechanical engineering, Applied mathematics

## Abstract

Based on the theory of equal base circle bevel gears, a new type of gear, the equal base circle herringbone curved bevel gear, is studied further to reduce the axial force problem of curved bevel gears. Using the principle of equal basis circle herringbone curved bevel gear as a basis, the tooth trace and the tool design of this gear were studied. Based on the principle of space meshing, the process of finger milling cutter enveloping machining gear was analyzed, and a tooth surface equation was calculated; This paper proposes two modeling methods: tooth trace modeling and tooth surface modeling. The gear model is consistent through the analysis and comparison of the two modeling methods. The axial force of the gear contact between the equal base circle herringbone curved bevel gear and Gleason are analyzed and compared, which verified the proper reduction of axial force.

## Introduction

Designed based on equal base circle theory, borrowed from herringbone tooth form, the equal base circle herringbone curved bevel gear is a new type of bevel gear. Both at home and abroad, research has yet to be conducted on this subject.

Compared with other bevel gears, straight bevel gears are simple to manufacture and have low axial force but low load-carrying capacity, poor transmission smoothness, and high noise level. Spiral bevel gears are a more complex gear to transmit power and motion in a vital power transmission system. The transmission process has the advantages of significant overlap, smooth transmission, high load-carrying capacity, and low noise, and it is suitable for high-speed gear transmission^1^. However, the axial force generated during the transmission of bevel gears affects the life of the gears. Yao^2^ analyzed axial force’s effect on the spiral bevel gear service life. The measures to improve the service life of bevel gears by the reasonable arrangement of axial force are proposed. Wang^3^ optimized the combination of the rotation of the spiral bevel gear, which can avoid the superposition of axial force, thus improving the pressure on the gear shaft and box of the reducer and improving the lives of the gear and bearing. Wu et al.^4^ provided a new analysis idea and balance scheme for the axial force produced in the working process of the double arc helical gear hydraulic pump, which can reduce the leakage owing to end clearance caused by the axial force and improve the volume efficiency of the gear hydraulic pump. Cao et al.^5^ provided that the arrangement of the reducer and the direction of rotation of the gears can be designed according to the operating characteristics of the machine so that the axial force of the large bevel gear and the axial force of the secondary shaft gear can be largely offset, thus greatly reducing the axial force on the bearings and improving the life of the bearings. Liu et al.^6^ analyzed that two gears’ spiral direction of the intermediate shaft is often the same so that its axial force could partly counteract each other. This will decrease the axial load of rolling bearings, rods, and gearboxes and prolong their service life. This will also provide a reference for similar gears’ design and installation. Zhu et al.^7^ provided that an adjustable axial thrust structure was developed to counteract the axial force of the gear. This paper proposes that the right-hand and left-hand axial forces of herringbone bevel gears can be canceled, thus reducing the combined axial force of bevel gears and improving gear life.

Based on the theory of equal base bevel gears, the motion equations of tooth traces and tool trajectories were established, and the selection principle of gear parameters was determined through the analysis of tooth line characteristics. According to the principle of spatial meshing, the tooth surface equation included in the tooth line is introduced, the geometric structure of the tooth surface is comprehensively analyzed, and the design of the herringbone curved bevel gear is realized. An equal base circle bevel gear is a curved tooth bevel gear unique to China, protected by Chinese patents. Its base radius at any cone distance is equal, and its tooth profile shape is the same, thus ensuring its processing tool, simple manufacturing process, and low cost of manufacture^8–10^. Cui et al.^11^, A new type of curved bevel gear was proposed, the equidistant line of tooth traces was calculated, and the particular equal base circle bevel gear analysis was carried out on the basis of this analysis of the geometric structure characteristics of this kind of bevel gear, the design of the tool, and the tooth shape error analysis, although the reference to the equal base circle bevel gear was not clear, in essence, it was also a study for the equal base circle bevel gear. Gong et al.^12,13^ made a profound and systematic study on the transmission principle, tooth surface geometry, tooth shape error, tooth modification, and meshing characteristics of the equal-base circle bevel gear by using modern calculation methods. Zhang et al. analyzed the tooth meshing characteristics, tooth geometry, and profile modification analysis of equal base circle bevel gear.^14^.

In response to the problem of the axial force of bevel gears, this paper proposes an innovative method of borrowing the herringbone tooth form from the tooth form of bevel gears to reduce the axial force of bevel gears. Herringbone curved bevel gear overcomes the shortcomings of the large axial force of the bevel gear. It is a bevel gear with a high degree of coincidence, strong bearing capacity, and long service life. Its processing equipment is simple, the processing cost is low, and the research has significant theoretical value and broad engineering application prospects.

## The principle of equal base circle herringbone curved bevel gears and tooth surface equations

This section starts from the basic principle of an equal base circle and studies its tooth trace characteristics and tool design. Based on the principle of spatial meshing, the process of machining herringbone curved bevel gears with finger-milling cutter envelopes is analyzed. Its tooth equations are accurately described to construct tooth surfaces and lay the foundation for analyzing tooth meshing characteristics.

### The tooth trace theory of equal base circle herringbone curved bevel gears

#### The principle of forming an equal base circle herringbone curved bevel gear

**Figure 1:**
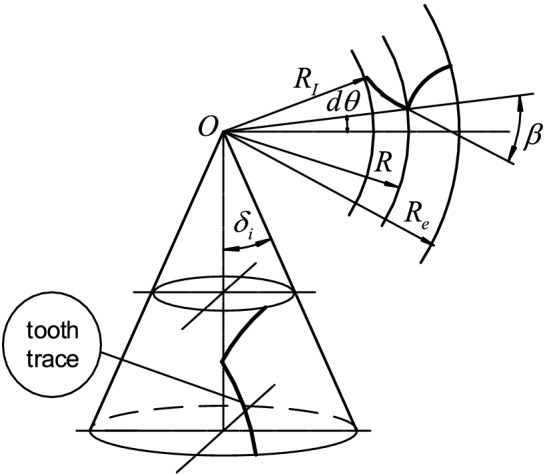
Geometry principle of the equal base circle herringbone curved bevel gear.

Figure 1 shows the tooth trace and geometric relationship between the reference cone and crown wheel plane of the equal base circle herringbone curved bevel gear.

During CNC machining, the tool and wheel blank motion is controlled to obtain a bevel gear with the same base circle radius at any cone distance—an equal base bevel gear^15–18^. The characteristics are derived as follows:

1 $$ r_{vb} = \frac{{zm_{t} \cos \alpha_{n} }}{{2\cos \delta \cos^{2} \beta }} = \frac{{zm_{te} \cos \alpha_{n} }}{{2\cos \delta \cos^{2} \beta e}} = cont $$

where $r_{vb}$ is the base circle radius of equivalent gear,$m_{t}$ is the transverse module, $m_{te}$ is the outer transverse module,$z$ is the tooth number of gear,$\delta$ is the indexing angle of bevel gear, $\beta$ and $\beta e$ are the helix angles of tooth trace corresponding to R and Re, R is the cone distance at an arbitrary point, Re is the cone distance of the big end,$\alpha_{n}$ is the normal pressure angle.

As given by Eq. (1):

2 $$ \cos^{2} \beta = \frac{R}{{\text{Re}}}\cos^{2} \beta e $$

The following information can be obtained from Eq. (2):

3 $$ \beta = \arccos \left( {\sqrt {\tfrac{R}{{{\text{R}}_{e} }}} \cos \beta_{e} } \right) $$

Equation (3) describes the relationship between the helical angle $\beta$ and the cone distance R.

#### The tooth trace equation of equal base circle herringbone bevel gears

**Figure 2:**
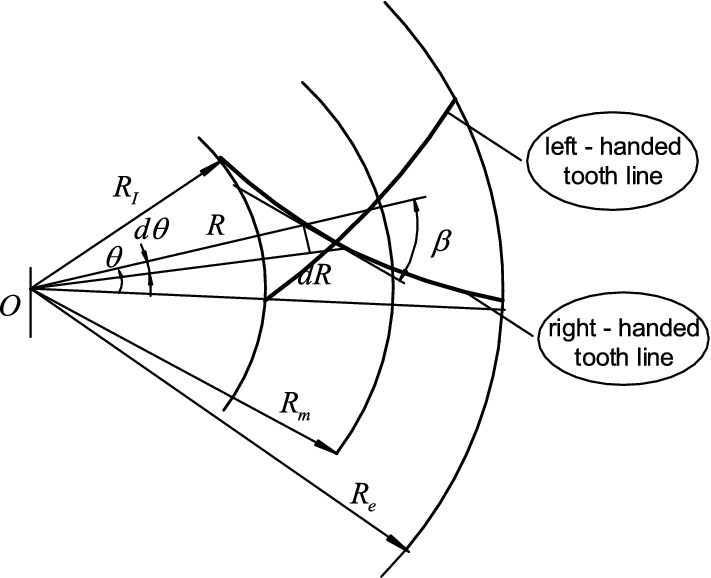
Theoretical tooth traces of the equal base circle herringbone curved bevel gear.

According to Fig. 2, the polar coordinate system is established with the crown wheel center as the pole and the ray at the big end of the gear as the polar axis. The differential equation of tooth trace can be derived as follows:

4 $$ Rd\theta = \pm \tan \beta dR $$

Equation (2) is differentiated and substituted into the equation above:

5 $$ d\theta = \pm \left( { - 2\tan^{2} \beta } \right)d\beta $$

The equation of the theoretical tooth trace of the herringbone curve bevel gear in the polar coordinate system is obtained by integrating Eq. (5).

6 $$ \theta = \pm 2\left( {\tan \beta - \beta } \right) + C $$

where C is the integration constant. “ + ” is for right-handed tooth trace. “−” is for left-handed tooth trace.

#### Equation of the tool path

**Figure 3:**
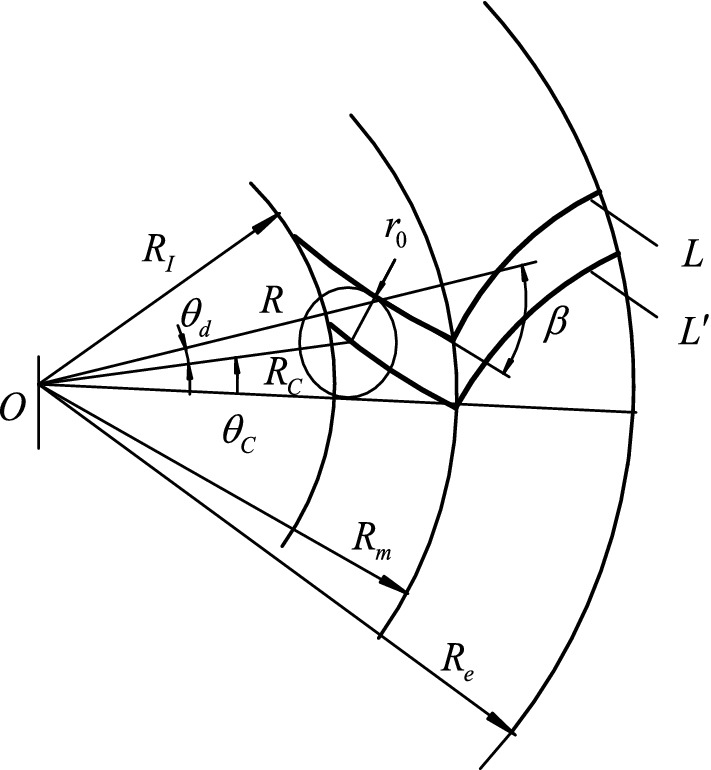
The relationship between tool path and tooth trace.

Figure 3 shows the relative position relationship between the finger milling cutter and tooth trace on the crown wheel plane. As the finger milling cutter moves along the equidistant line $L^{\prime }$ (tool path) of the theoretical tooth trace $L$, the tool surface envelopes the actual tooth line of equal base circle herringbone curved bevel gears without modification. In polar coordinates, the equation of tool center trajectory is:

7 $$ R_{c} = \sqrt {R^{2} + r_{0}^{2} \pm 2Rr_{0} {\text{Sin}} \beta } $$

8 $$ \theta_{d} = \arcsin \left( {\frac{{r_{0} }}{{R_{c} }}\cos \beta } \right) $$

9 $$ \theta_{c} = \pm 2(\tan \beta - \beta ) + \theta_{d} $$

where $r_{0}$ is the distance between the theoretical tooth trace and its equidistant line, determined by the cutter size.

In Eq. (7), “ + ” is for the concave tooth surface, and “−” is for the convex tooth surface.

In Eq. (8), $\theta_{d}$ is the angle between the polar diameter R of the theoretical tooth line and the polar diameter $R_{c}$ of the tool's center trajectory at the corresponding point.

In Eq. (9), “ + ” is for right-handed concave tooth surface or left-handed convex tooth surface, and “−” is for left-handed concave tooth surface or right-handed convex tooth surface.

### The designing of the finger milling cutter

#### The finger milling cutter shaft section

To avoid interference with the cutter when machining herringbone curved bevel gear with a finger milling cutter. Making sure that: based on the tooth width of the small end of the gear, the graduated arc tooth thickness of the finger milling cutter must be less than the tooth width of the small end (the cutter radius shall be less than half of the tooth width). Therefore, the cutter must be designed according to the profile of the gear with specific relationship cone distance parameters, i.e. $R_{j} > R_{i}$.

As a result of Eq. (1), the modulus and the number of teeth of equivalent gear at cone distance $R_{j}$ are as follows:

10 $$ m_{nj} = m_{te} \varphi_{R}^{\frac{3}{2}} \cos \beta e $$

11 $$ z_{vj} = \frac{z}{{\psi_{R}^{\frac{3}{2}} \cos \delta \cos^{3} \beta_{e} }} $$

12 $$ r_{v} = 0.5m_{nj} .z_{vj} $$

where $\psi_{R}$ is the cone distance coefficient. $r_{v}$ is the radius of the standard pitch circle for equivalent gear.

The tool radius is less than half of the width of the small end groove, thus meeting the requirements:

13 $$ r_{0} < \frac{{\pi m_{nj} }}{4} $$

**Figure 4:**
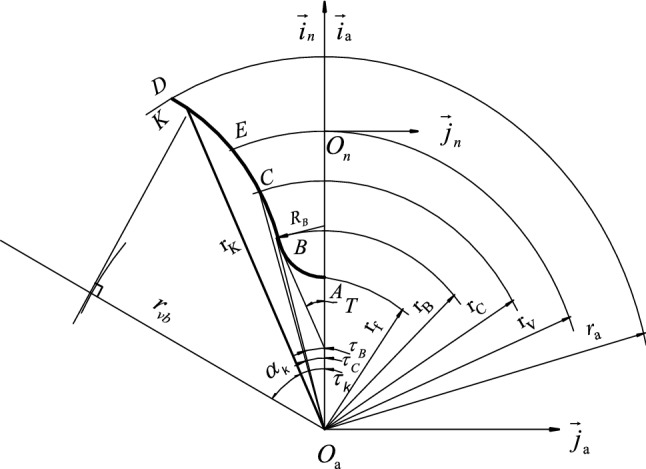
Cutter shaft section of finger milling cutter.

According to the requirements of gear transmissions, to avoid interference between the working part of the tooth profile and the meshing tooth profile during machining, a finger milling cutter's shaft profile must be designed according to the tooth profile of an equivalent straight cylindrical gear. Figure 4 shows the profile of the finger milling cutter.

In Fig. 4, $\sigma_{{\text{a}}} :\left[ {O_{{\text{a}}} {-\!\!-}\overrightarrow {i}_{{\text{a}}} ,\overrightarrow {j}_{{\text{a}}} } \right]$ is the axis sectional coordinate system of the finger milling cutter. $\sigma_{n} :\left[ {O_{n} {-\!\!-}\overrightarrow {i}_{n} ,\overrightarrow {j}_{n} } \right]$ is the auxiliary coordinate system of the tool. AB is the arc. CD is the involute of equivalent gear profile at the design tool. BC is the straight line connecting AB and CD.

For cutter profiles in this paper, only the CD segment involute tooth profile is used.

#### The calculation of the tool special point C

Point C is the tangent point between the tool-tip path’s extended involute and the tooth profile's involute when the rack machines the spur gear. The coordinates of point C in the tool auxiliary coordinate system are as follows:

14 $$ \left\{ \begin{gathered} x_{c} = r_{c} \cos \tau_{c} \hfill \\ y_{c} = - r_{c} \sin \tau_{c} \hfill \\ \end{gathered} \right. $$

where

15 $$ r_{c} = \sqrt {\left( {r_{v} \sin \alpha_{n} - \frac{ha}{{\sin \alpha_{n} }}} \right)^{2} + (r_{v} \cos \alpha_{n} )^{2} } $$

16 $$ \left\{ \begin{gathered} h_{a} = m_{nm} \hfill \\ \tau_{c} = \frac{\pi }{{2z_{vj} }} - (\tan \alpha_{n} - \alpha_{n} ) + (\tan \alpha_{c} - \alpha_{c} ) \hfill \\ \alpha_{c} = \arccos \left( {\frac{{r_{vb} }}{{r_{c} }}} \right) \hfill \\ \end{gathered} \right. $$

In Eq. (16), $m_{nm}$ is the normal modulus.$h_{a}$ is tooth tip height.$\alpha_{n}$ is the normal pressure angle.$\alpha_{c}$ is the pressure angle at point c.

#### The actual profile equation of a finger milling cutter

According to the axis sectional coordinate system of the finger milling cutter, the CD segment of the cutter’s axis profile is equivalent gear profile asymptote. For gear transmission requirements, when profiling herringbone curved bevel gears, the CD section of the working part of the gear profile should be high enough considering the complete machining of the tooth top of the gear.

The profile equation of the CD segment in the coordinate system $\sigma_{{\text{a}}}$ is:

17 $$ \left\{ \begin{gathered} x_{k} = r_{k} \cos \tau_{k} \hfill \\ y_{k} = - r_{k} \sin \tau_{k} \hfill \\ \end{gathered} \right. $$

The profile equation of the CD segment in the coordinate system $\sigma_{n}$ is:

18 $$ \left\{ \begin{gathered} x_{n} = r_{k} \cos \tau_{k} - r_{v} \hfill \\ y_{n} = - r_{k} \sin \tau_{k} \hfill \\ \end{gathered} \right. $$

**Figure 5:**
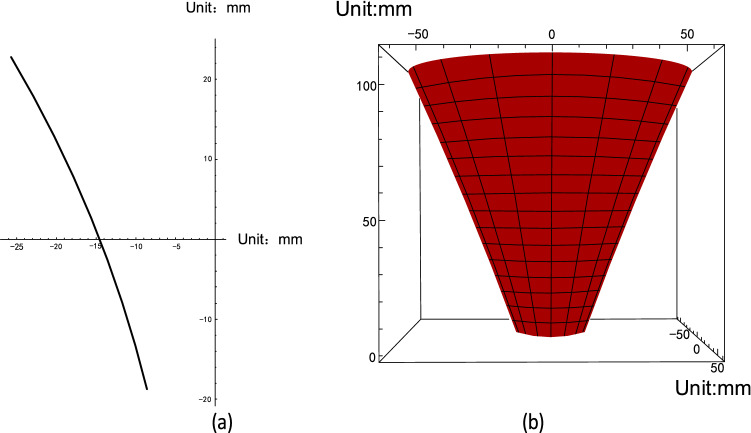
Two-dimensional and three-dimensional sections of the cutter CD section.

The two-dimensional and three-dimensional section of the cutter CD section is shown in Fig. 5.

### The tooth surface equation of equal base circle herringbone bevel gears

#### The cutting coordinate system and change matrix

**Figure 6:**
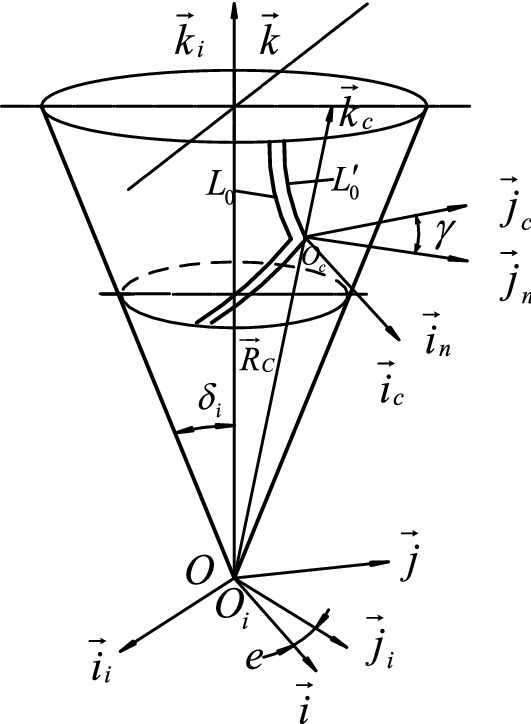
The gear-cutting coordinate system.

In the finger cutter milling process, the tool makes a uniform linear motion from the gear's big end to the gear's small end along the reference cone generatrix. Meanwhile, the wheel blank makes a variable speed rotary motion according to a specific law, and the relative movement between the tool surface of the wheel blank and the wheel blank envelops the tooth surface of the herringbone curved bevel gears. The tool surface is rotary, and there is a contact line with the tooth surface every instant during the motion. All of the contact lines are assembled and enveloped in the tooth surface. A herringbone curved bevel gear’s tooth surface equation and geometric characteristics are determined by the cutter’s geometric parameters and the relative motion between the cutter and the wheel blank. The gear-cutting coordinate system is shown in Fig. 6.

In Fig. 6, $\sigma_{i} :\left( {O_{i} {-\!\!-}\overrightarrow {{i_{i} }} ,\overrightarrow {{j_{i} }} ,\overrightarrow {{k_{i} }} } \right)$ is the wheel blank coordinate system. $\sigma_{c} :\left( {O_{c} {-\!\!-}\overrightarrow {{i_{c} }} ,\overrightarrow {{j_{c} }} ,\overrightarrow {{k_{c} }} } \right)$ is the tool coordinate system. $\sigma :\left( {O{-\!\!-}\overrightarrow {i} ,\overrightarrow {j} ,\overrightarrow {k} } \right)$ is the fixed coordinate system.

The tool is moved from the tool coordinate system to the wheel blank coordinate system using three right-handed rectangular coordinate systems. As shown in Fig. 6: during gear cutting, the tool coordinate system is always parallel to the cone generatrix, and the $\overrightarrow {{j_{c} }}$ axis of the tool coordinate system $\sigma_{c}$ are parallel to the $\overrightarrow {j}$ axis of the fixed coordinate system $\sigma$. The tool coordinate system $\sigma_{c}$ is first translated along the $\overrightarrow {{k_{c} }}$ axis by the distance $R_{c}$, so that the origin of the tool coordinate system coincides with the origin coordinate of the wheel blank coordinate system and the origin of the fixed coordinate system. This auxiliary coordinate system is defined as $\sigma_{t}$. Auxiliary coordinate system $\sigma_{t}$ is then rotated by an angle $\delta$ around the $\overrightarrow {j}$ axis to change under the fixed coordinate system and the tool equation under the fixed coordinate system is rotated by an angle e around the $\overrightarrow {k}$ axis to change under the wheel blank coordinate $\sigma_{i}$, resulting in the tool coordinate system $\sigma_{c}$ moves to the tool surface under the wheel blank coordinate system $\sigma_{i}$.

The coordinate transformation is as follows:

19 $$ M_{ic} = \, M_{io} \, \cdot M_{ot} \, \cdot M_{tc} $$

20 $$ M_{tc} = \left( {\begin{array}{*{20}c} 1 & 0 & 0 & 0 \\ 0 & 1 & 0 & 0 \\ 0 & 0 & 1 & {R_{c} } \\ 0 & 0 & 0 & 1 \\ \end{array} } \right) $$

21 $$ M_{ot} = \left( {\begin{array}{*{20}c} {\cos \delta } & 0 & {\sin \delta } & 0 \\ 0 & 1 & 0 & 0 \\ { - \sin \delta } & 0 & {\cos \delta } & 0 \\ 0 & 0 & 0 & 1 \\ \end{array} } \right) $$

22 $$ M_{io} = \left( {\begin{array}{*{20}c} {\cos e} & { - \sin e} & 0 & 0 \\ {\sin e} & {\cos e} & 0 & 0 \\ 0 & 0 & 1 & 0 \\ 0 & 0 & 0 & 1 \\ \end{array} } \right) $$

The vector relationship between $\sigma$ and $\sigma_{i}$ origin is:

23 $$ \overrightarrow {{R_{c} }} = R_{c} (\sin \delta_{i} \overrightarrow {i} + \cos \delta_{i} \overrightarrow {k} ) $$

where e is the included angle between the $\overrightarrow {{j_{i} }}$ axis of the wheel blank coordinate system and the $\overrightarrow {i}$ axis of the fixed coordinate system. The derivation of $e$ is based on the equal relationship between the counter-rolling arc length of the coning bus tooth trace and the tooth trace in the crown wheel plane. Here is how it is calculated:

24 $$ r \cdot e = r_{c} \cdot \theta_{c} \Rightarrow \frac{{\theta_{c} }}{{\frac{r}{{r_{c} }}}} \Rightarrow e = \frac{{\theta_{c} }}{{\sin \delta_{i} }} $$

#### The tool surface of revolution and its unit tangent vector and normal vector

The rotary surface equation of the tool profile CD is:

25 $$ \overrightarrow {r}^{(c)} = x_{c} \overrightarrow {{i_{c} }} + y_{c} \overrightarrow {{j_{c} }} + z_{c} \overrightarrow {{k_{c} }} $$

where

26 $$ \left\{ \begin{gathered} x_{c} = r_{k} \cos \tau_{k} - r_{v} \hfill \\ y_{c} = - r_{k} \sin \tau_{k} \cos \gamma \hfill \\ z_{c} = r_{k} \sin \tau_{k} \sin \gamma \hfill \\ \end{gathered} \right. $$

The tool rotary surface is parameterized with $\gamma$ and $\alpha_{k}$ as the parameter variable. The direction of its parametric curve is its principal direction, and the corresponding unit tangent vectors of the principal direction are $\overrightarrow {g}_{i1}^{(c)}$ and $\overrightarrow {g}_{i2}^{(c)}$, respectively.

To ensure that the normal vector at the point of engagement of the teeth of the gear and pinion face is the same for the analysis of the meshing characteristic. Taking the positive direction of the parameter curve $\alpha_{k}$, the first principal direction $\overrightarrow {g}_{11}^{(c)}$ of the pinion cutter surface. Taking the negative direction of the parameter curve $\alpha_{k}$, the first principal direction $\overrightarrow {g}_{21}^{(c)}$ of the gear cutter surface. The second principal directions $\overrightarrow {g}_{12}^{(c)}$, $\overrightarrow {g}_{22}^{(c)}$ of the tool surfaces of the gear and the pinion are the positive directions of the parameter curve $\gamma$,$\overrightarrow {g}_{i1}^{(c)}$ and $\overrightarrow {g}_{i2}^{(c)}$ are the unit tangent vectors of the tool surface.

27 $$ \overrightarrow {g}_{i1}^{(c)} = ( - 1)^{(i - 1)} \frac{{D_{1} \overrightarrow {{i_{c} }} - D_{2} \cos \gamma \overrightarrow {{j_{c} }} + D_{2} \sin \gamma \overrightarrow {{k_{c} }} }}{{\sqrt {D_{1}^{2} + D_{2}^{2} } }} $$

where

28 $$ D_{1} = r_{vb} \cos \left( {\frac{\pi }{{2z_{vj} }} + \alpha_{n} + \tan \alpha_{k} - \tan \alpha_{n} } \right)\sec (\alpha_{k} )^{2} \tan \alpha_{k} $$

29 $$ D_{2} = r_{vb} \sec \left( {\alpha_{k} } \right)^{2} \sin \left( {\frac{\pi }{{2z_{vj} }} + \alpha_{n} + \tan \alpha_{k} - \tan \alpha_{n} } \right)\tan \alpha_{k} $$

30 $$ \overrightarrow {g}_{i2}^{(c)} = \sin \gamma \overrightarrow {{j_{c} }} + \cos \gamma \overrightarrow {{k_{c} }} $$

The normal vector of the tool surface is:

31 $$ \begin{gathered} \overrightarrow {n}_{i}^{(c)} = \overrightarrow {g}_{i1}^{(c)} \times \overrightarrow {g}_{i2}^{(c)} = ( - 1)^{(i - 1)} \frac{{\left( { - D_{2} \overrightarrow {i}_{c} - D_{1} \cos \gamma \overrightarrow {j}_{c} + D_{1} \sin \gamma \overrightarrow {k}_{c} } \right)}}{{\sqrt {D_{1}^{2} + D_{2}^{2} } }} \hfill \\ i = 1,2 \hfill \\ \end{gathered} $$

According to the tangent vector’s setting, the tool’s normal vector is directed from the tool entity to the empty field. The normal vector of the tooth being machined is directed from the empty field to the blank wheel entity when machining the face of the pinion.

When cutting the face of the gear, the normal vector of the tool surface points from the empty field to the entity of the tool, and the normal vector of the face of the wheel blank points from the entity to the empty field.

A common normal vector at the meshing point of the two-wheel surfaces points from the gear surface to the pinion surface.

#### The relative speed of contact point between tool surface and tooth surface

During the cutting process, the tool moves from the large end of the wheel blank to the small end at a constant speed of $\frac{{dR_{c} }}{dt}$. In $\sigma_{c}$:

32 $$ \overrightarrow {{v_{c} }}^{(c)} = \frac{{d\overrightarrow {R}_{{_{C} }} }}{dt} = \frac{{dR_{c} }}{dt}\overrightarrow {k}_{c} $$

when the tool moves at speed $\frac{{dR_{c} }}{dt}$, the crown wheel makes a rotary movement at angular speed $\omega_{c}$, ensuring that the tool center trajectory is an isometric line of the tooth line. From Eq. (9), it is deduced that the crown wheel angular speed $\omega_{c}$ is:

33 $$ \omega_{c} = \frac{{d\theta_{c} }}{dt} $$

Based on the rolling relationship between the tooth trace of the crown wheel plane and the reference cone and Eq. (33), when the tool is at $R_{c}$, the instantaneous angular velocity of the wheel blank is:

34 $$ \overrightarrow {\omega }^{(i)} = \frac{{\frac{{d\theta_{c} }}{dt}}}{{\sin \delta_{i} }}\overrightarrow {k} = A\frac{{dR_{c} }}{dt}\overrightarrow {k} $$

where

35 $$A = \frac{{\left( { \pm \frac{\tan \beta }{{R\frac{{dR_{c} }}{dR}}} \pm \frac{{d\theta_{d} }}{{dR_{c} }}} \right)}}{{\sin \delta_{i} }} $$

In Eq. (35), the first “ + ” is for right-handed tooth trace, and “−” is for left-handed tooth trace. The second “ + ” is for right-handed concave tooth surface or left-handed convex tooth surface, and “−” is for left-handed concave tooth surface or right-handed convex tooth surface.

The velocity of a toothed surface is expressed under the tool coordinate system $\sigma_{c}$.

36 $$ \overrightarrow {v}_{c}^{\left( i \right)} = \left[ {M_{CO} } \right]\left( {\overrightarrow {\omega }^{(i)} \times \overrightarrow {{r_{0} }}^{(i)} } \right) $$

where

37 $$\overrightarrow {{r_{0} }}^{(i)} = \overrightarrow {{R_{c} }} + \left[ {M_{OC} } \right]\overrightarrow {{r_{0} }}^{(i)} $$

According to Eq. (36) and Eq. (37), the relative velocity of the tool surface to the tooth surface contact point is:

38 $$ \overrightarrow {v}_{c}^{(ci)} = \overrightarrow {v}_{c}^{(c)} - \overrightarrow {v}_{c}^{(i)} = A\left\{ - \cos \delta_{i} y_{c} \overrightarrow {i}_{c} { + }[\cos \delta_{i} x_{c} + \sin \delta_{i} \left( {z_{c} + R_{c} } \right)]\overrightarrow {j}_{c} - \sin \delta_{i} y_{c} \overrightarrow {k}_{c} \right\} \frac{{dR_{c} }}{dt} $$

#### The tooth surface equation

At the conjugate contact line, the tool surface and the tooth surface of the wheel blank should satisfy the mesh equation in the coordinate system $\sigma_{c}$:

39 $$ \overrightarrow {n}_{i}^{(c)} \cdot \overrightarrow {v}_{c}^{(c)} = 0 $$

Obtained by Eqs. (31), (38) into Eq. (39):

40 $$ tg\gamma = A\cos \delta \left( {\frac{{D_{1} }}{{D_{2} }}\left( { - r_{k} \sin \tau_{k} } \right) - \left( {r_{k} \cos \tau_{k} - r_{v} + R_{c} \tan \delta } \right)} \right) $$

Given a different value $\alpha_{k}$, one can obtain a contact line $\overrightarrow {r}^{(c)} \left( {\alpha_{k} ,R} \right)$ between the tool surface and the tooth surface at that instant. For different tool positions at $R_{c} \left( R \right)$, an instantaneous family of contact lines $\left\{ {\overrightarrow {r}^{(c)} \left( {\alpha_{k} ,R} \right)} \right\}$ is obtained, which forms the gear tooth surface in the wheel blank coordinate system $\sigma_{i}$.The tooth surface equation is as follows:

41 $$ V_{ri} { = }M_{io} \cdot M_{ot} \cdot M_{tc} \cdot \overrightarrow {r}^{{_{\left( c \right)} }} $$

#### Create the tooth surfaces

**Figure 7:**
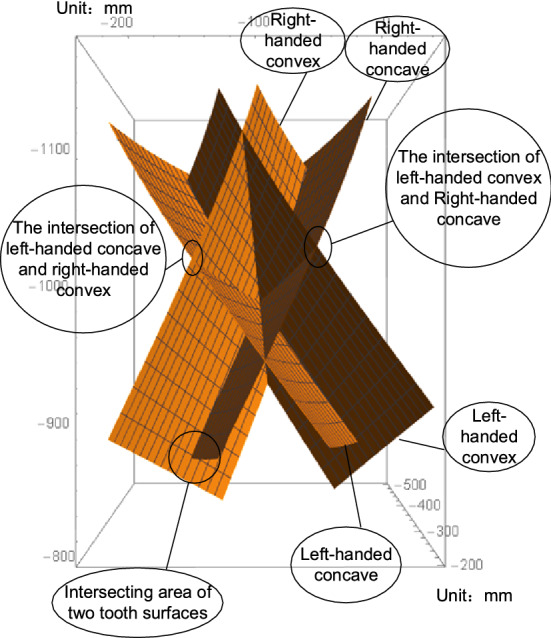
Tooth surfaces.

Generate tooth surfaces, as shown in Fig. 7, and tooth surface point coordinates according to a certain rule for export.

## Modeling

In order to verify the correctness of the calculation model, this section proposes two modeling methods: tooth trace modeling and tooth surface modeling, using the gear as an example, and a comparative analysis of the gear models derived from these two modeling methods.

### Modeling (according to the tooth traces, using the gear as an example)

#### Importing tooth trace points

In Eqs. (7), (8), and (9), R is the independent variable in the above equation. R is taken from the cone distance of the small end $R_{i}$ to $R_{e}$ in turn +5 and substituted into the above equation to find out the tooth trace points on the left-hand and right-hand concave-convex surfaces of the gear and pinion, respectively, after a coordinate transformation, the tooth trace on the plane of the crown wheel is transformed from under the plane coordinate system to under the spatial coordinate system, and the three-dimensional coordinates are as follows:

42 $$ x = - R_{c} \sin \delta \cos e $$

43 $$ y = R_{c} \sin \delta \sin e $$

44 $$ z = - R_{c} \cos e $$

Create the .txt file from the tooth trace points and save them.

In the 3D software environment, create a wheel blank sketch based on the gear parameters; the rotate command generates a 3D model of the wheel blank. Table 1 shows the gear parameters.

**Table 1:** The gear parameters.

Geometric parameters	Pinion	Gear
Number of teeth	26	77
Outer transverse module (mm)	30	30
Outer spiral angle (°)	17	17
Face width (mm)	310	310
Normal pressure (°)	20	20

Select the right-handed concave tooth trace and the left-handed concave tooth trace into the 3D software, respectively. The right-convex tooth trace and the left-convex tooth trace, the right-convex tooth trace, and the left-convex tooth trace each have an intersection point, respectively, with these two intersections as reference points.

**Figure 8:**
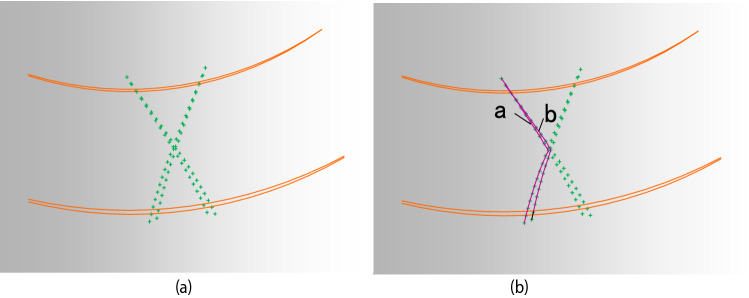
Tooth trace points.

A curve connecting the tooth trace points of the right-handed convex part below the intersection point and the left-handed concave tooth line points above the intersection point is used and defined as a. A curve connecting the line points of the right-handed concave part below the intersection with the line points of the left-handed convex part above the intersection and defined as b. This is shown in Fig. 8.

#### Surface of a building

1. A straight line perpendicular to the reference cone is established by a point defined as axis c.
2. Create a face perpendicular to the tooth trace and define it as $\Sigma 1$.
3. Create a face perpendicular to axis c and define it as $\Sigma 2$.
4. The line of $\Sigma 1$ ∩ $\Sigma 2$ is defined as axis d.
5. Lines c and d define a face and define it as $\Sigma 3$.

#### Create a tool

The tooth shape with a different module, different number of teeth, and the same diameter of the graduation circle is generated. In order to complete the machining, the tooth with the smallest module is taken as the finger cutter with the involute profile. The number of tool teeth is rounded to ensure a low normal modulus.

#### Tool for importing

**Figure 9:**
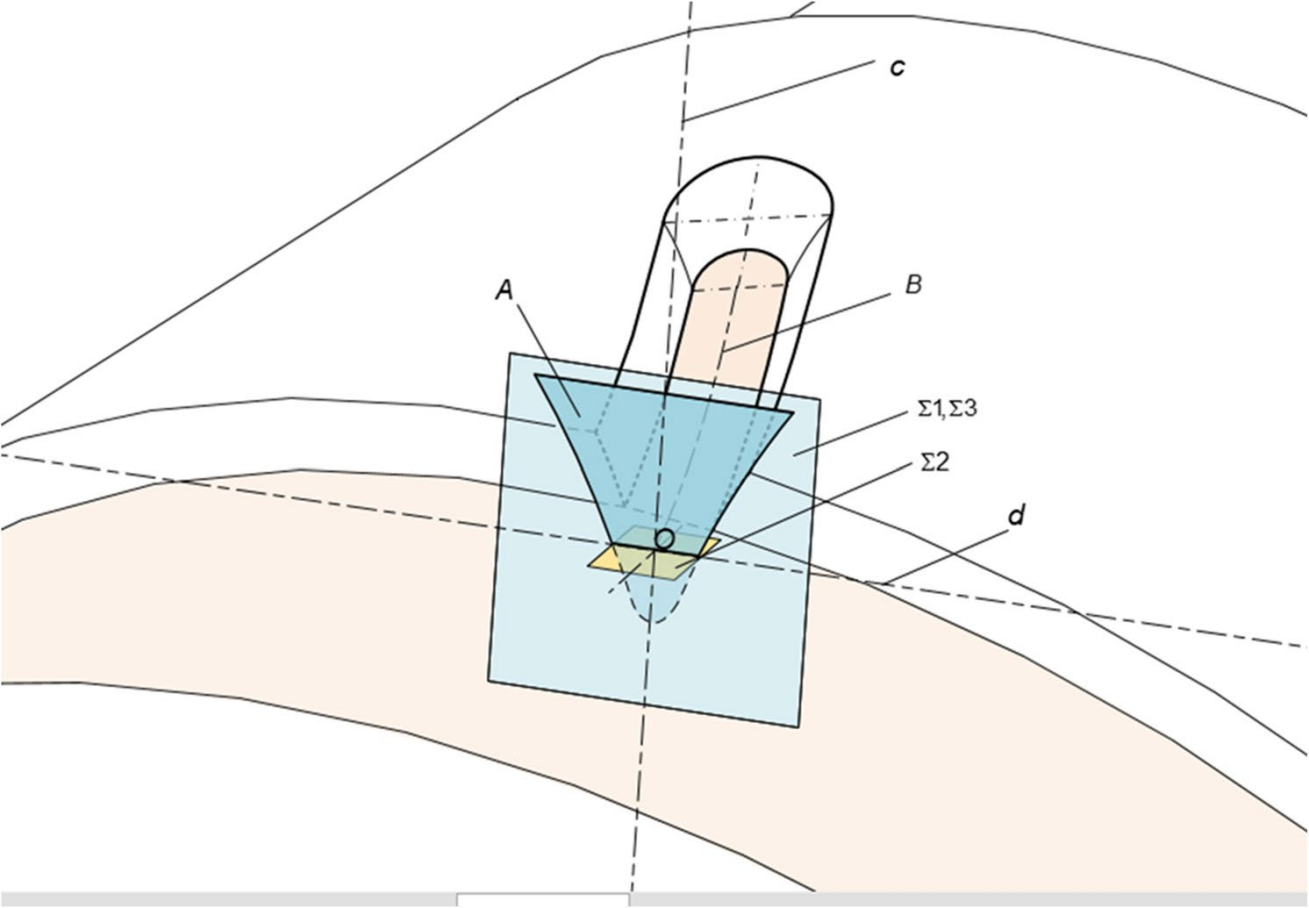
Sweeping the conical surface.

Select the generated tooth file to import into the 3D modeling environment, move the tool to $\Sigma 3$ and make the center line of the tool coincide with the center line of $\Sigma 3$. As shown in Fig. 9.

#### The generation of a model

**Figure 10:**
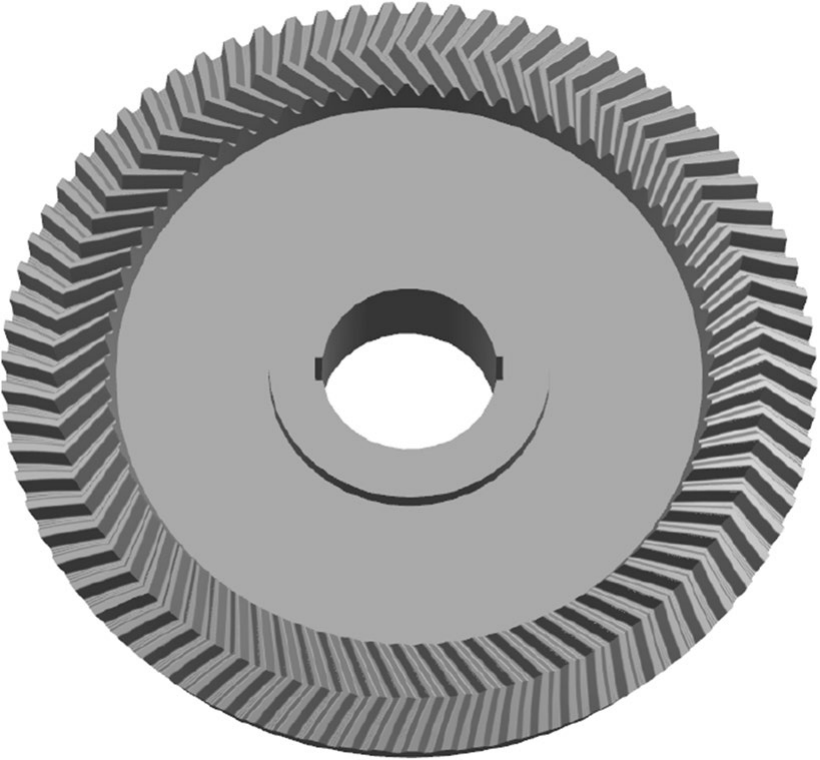
The gear model.

**Figure 11:**
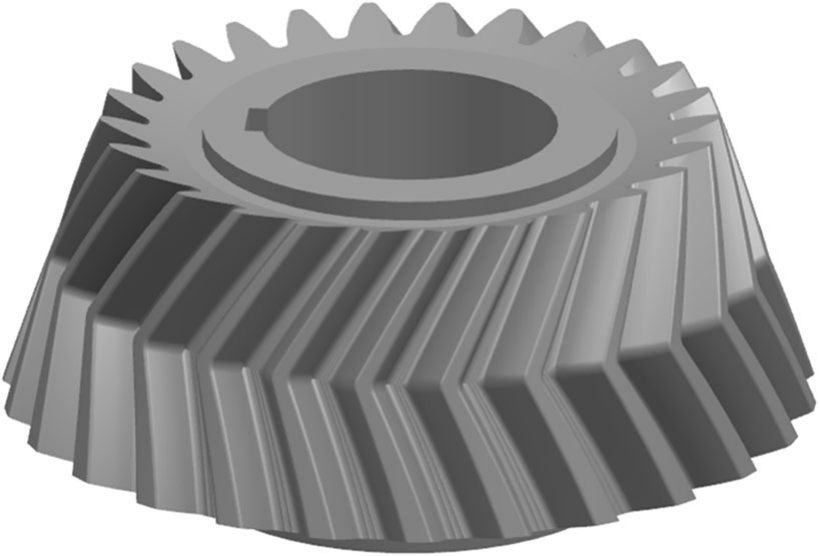
The pinion model.

As shown in Fig. 9. With B as the guideline and the reference cone as the guide surface, the cutting tool A sweeps the wheel blank to generate the individual tooth profile. Finally, the gear model is obtained from the circular array. The gear model is shown in Fig. 10. For the pinion, the same method is used as above. The pinion model is shown in Fig. 11.

### Modeling (according to the tooth surfaces , using the gear as an example)

**Figure 12:**
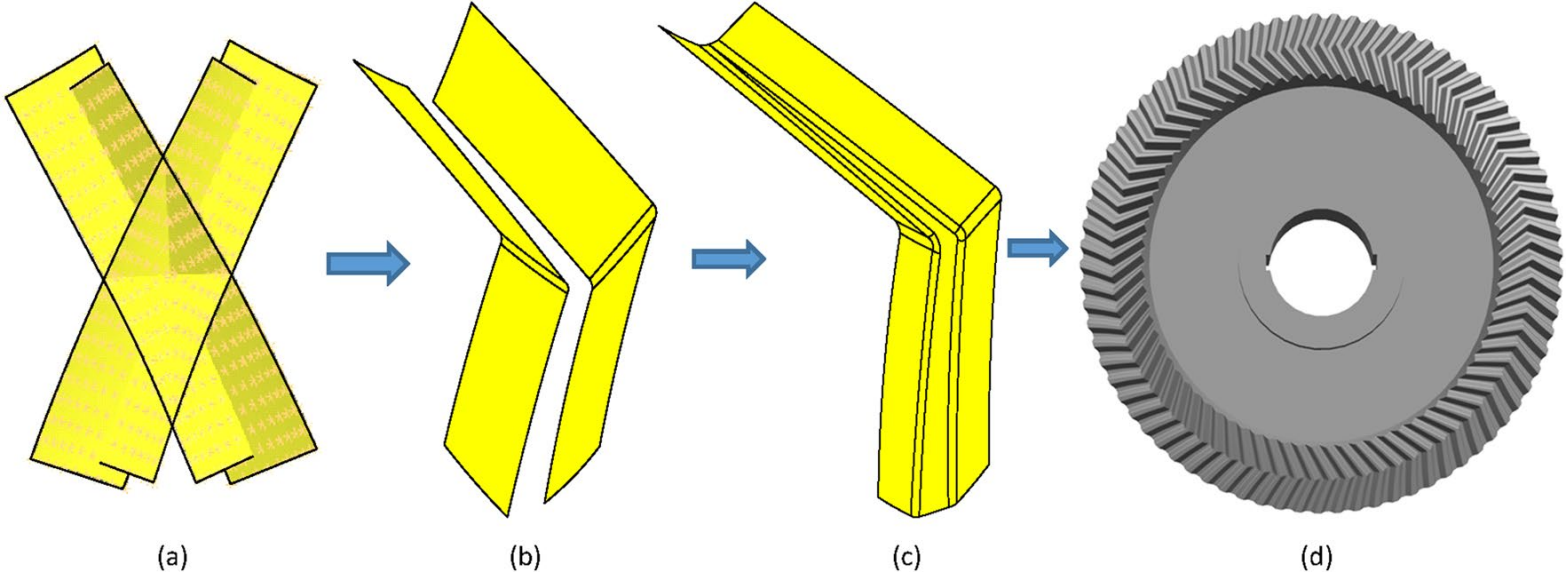
The gear model.

Import the calculated tooth point coordinates of the left-handed concave and convex surfaces and the right-handed concave and convex surfaces into a 3D modeling environment in a .dat format file, respectively. The gear's concave and convex tooth surfaces are then generated. To create a complete tooth groove, the concave and convex tooth surfaces are trimmed and connected to the top of the concave and convex surfaces, and then array the tooth grooves by the number of gear teeth to generate an accurate 3D model of the gear of the herringbone bevel gear. The gear model is shown in Fig. 12. Here’s how:

As before, the pinion is modeled in the same way.

**Figure 13:**
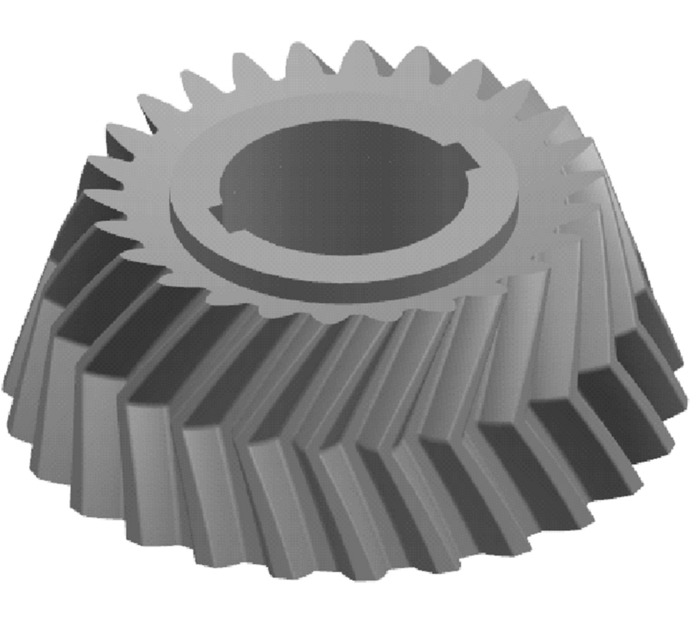
The pinion model.

**Figure 14:**
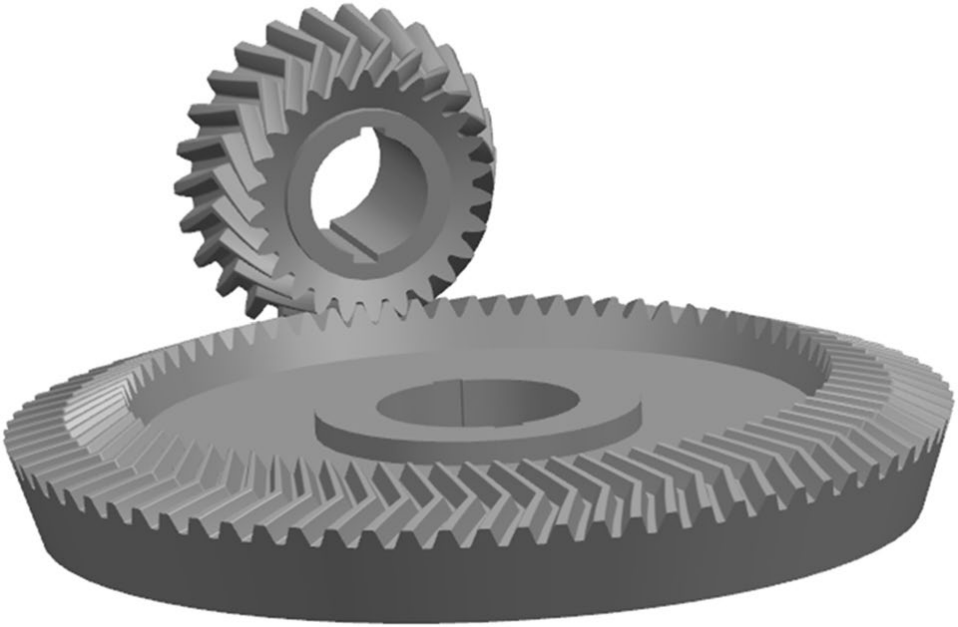
Assemble the model.

The pinion model is shown in Fig. 13. Assemble the model is shown in Fig. 14.

### Comparison of tooth trace modeling and tooth surface modeling

**Figure 15:**
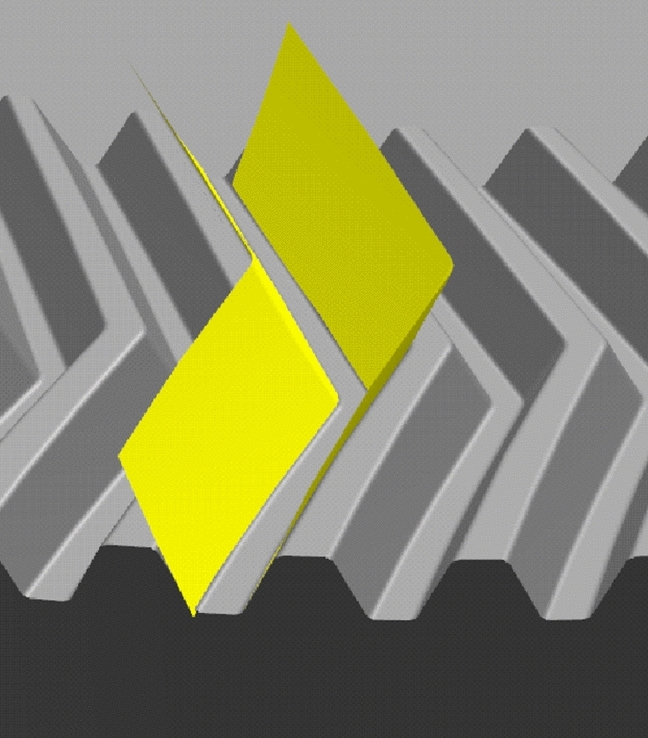
The comparison result.

The tooth surfaces generated by the tooth surface modeling are imported into the gear model generated by the tooth trace modeling. The tooth surfaces fit the concave and convex surfaces of the gear model generated by the tooth trace modeling. The comparison result is shown in Fig. 15.

## An analysis of the axial force of the equal base circle herringbone curved bevel gear contact

At the nodes, the gear and pinion mesh, and the load distribution along the tooth width is equivalently transformed into a normal force concentrated at the midpoint of the tooth width, which is usually decomposed into three mutually perpendicular components: tangential force, radial force and axial force. Table 2 shows the force calculation formula for the spiral bevel gear^3^.

**Table 2:** The force calculation formula for the spiral bevel gear.

Direction of turning	Left-handed	Right-handed	Left-handed	Right-handed
Direction of rotation	Clockwise	Anticlockwise	Anticlockwise	Clockwise
Tangential force	$F_{t} = \frac{2000T}{d}$			
Radial force	$F_{r} = \frac{{F_{t} }}{\cos \beta }\left( {\tan \alpha_{n} \sin \delta - \sin \beta \cos \delta } \right)$		$F_{r} = \frac{{F_{t} }}{\cos \beta }\left( {\tan \alpha_{n} \sin \delta - \sin \beta \cos \delta } \right)$	
Axial force	$F_{a} = \frac{{F_{t} }}{\cos \beta }\left( {\tan \alpha_{n} \sin \delta - \sin \beta \cos \delta } \right)$		$F_{a} = \frac{{F_{t} }}{\cos \beta }\left( {\tan \alpha_{n} \sin \delta + \sin \beta \cos \delta } \right)$	

Where: T is the Torque applied by the active wheel.

**Figure 16:**
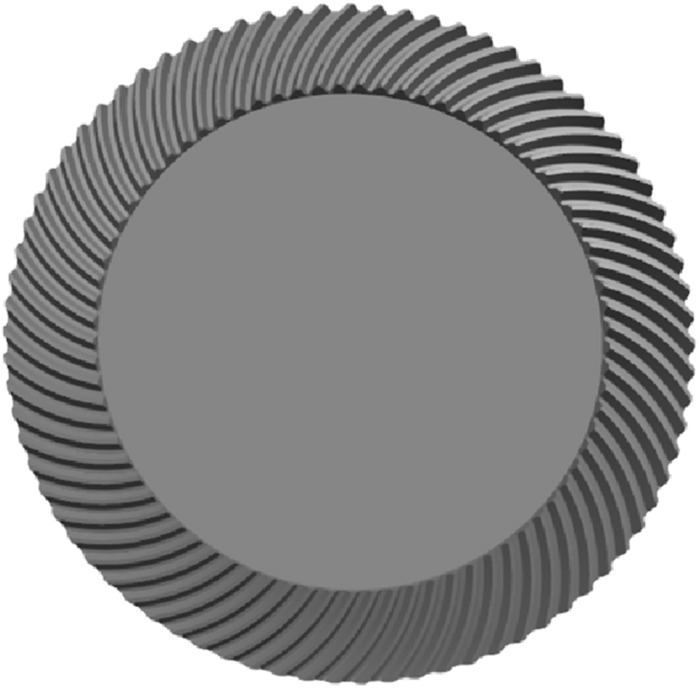
The gear model of Gleason.

**Figure 17:**
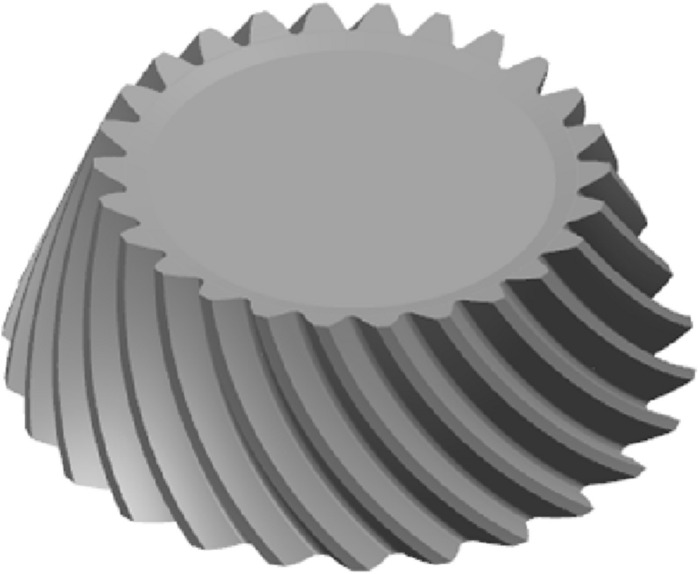
The pinion model of Gleason.

**Figure 18:**
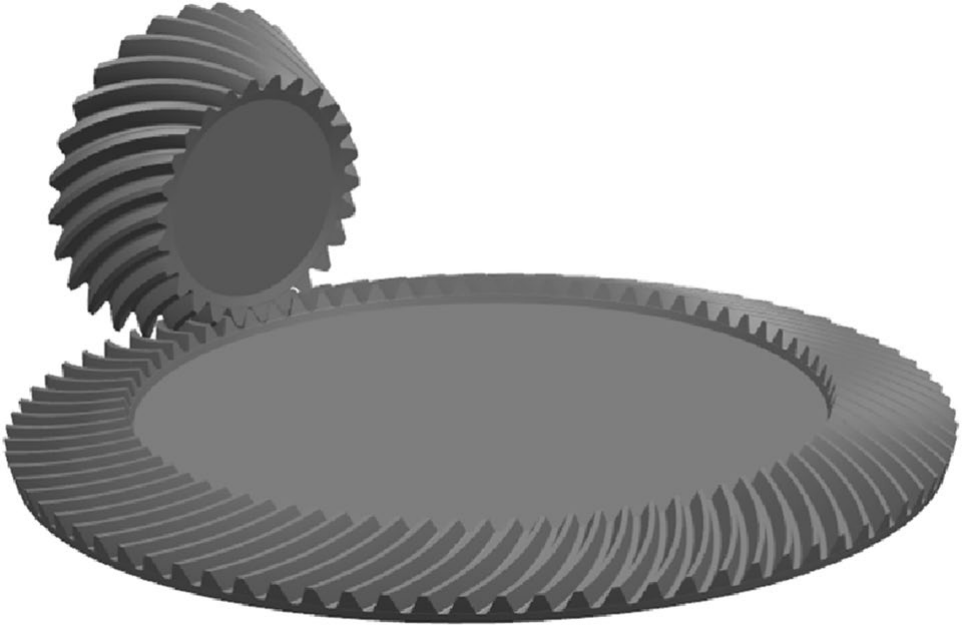
Assemble the model of Gleason.

The ratio analysis is conducted with Gleason to ensure the accuracy of the results. The geometry of Gleason is shown in Figs. 16, 17 and 18, and the parameters of Gleason are shown in Table 3.

**Table 3:** The parameters of the Gleason.

Geometric parameters	Pinion	Gear
Number of teeth	26	77
Outer transverse module (mm)	30	30
Spiral angle (°)	35	35
Face width (mm)	310	310
Normal pressure (°)	20	20

The equal base circle herringbone curved bevel gear can be seen as two spiral bevel gears, assuming that the helical angle between the right-handed bevel gear and the left-handed bevel gear is equal. Based on the formula in Table 2, it can be seen that the right-handed axial and left-handed axial forces significantly offset the trend. As the Gleason rotation is constant, the axial force has no offset trend, so we can see that the equal base circle herringbone curved bevel gear has a smaller force than the Gleason. The tangential force, radial force, axial force, and values cannot be calculated due to differences in distribution torque and knot diameter at each node(at different cone distances, the end face modulus varies). It requires a static contact simulation analysis to verify its computational analysis.

As the gear transmission process proceeds, the changing contact state of the gear will produce a complex nonlinear problem, and the finite element method is an effective way to solve nonlinear contact problems. Using the finite element analysis software, import the assembled 3D model into the static finite element analysis interface, define the contact pair, and divide the mesh. In the static analysis, assuming that the gear and pinion are in contact instantaneously, the pinion doesn't move, the gear rotates at a given angular speed, and The constraint is applied to the pinion bore surface. The inner gear shaft bore face is used for 2000 nm torque^19,20^.

Newton's third law states that tangential force X, radial force Y, and axial force Z are obtained through a supporting reaction force.

Tables 4 and 5 show the results of the analysis.

**Table 4:** Results of equal base circle herringbone curved bevel gears.

Maximum value over time	
X axis	1877.3 N
Y axis	25.3 N
Z axis	− 54.774 N
Total	1935.5 N

Minimum value over time	
X axis	400.16 N
Y axis	− 310.76 N
Z axis	− 354.16 N
Total	403.92 N

**Table 5:** Results of Gleason.

Maximum value over time	
X axis	2250.7 N
Y axis	− 411.03 N
Z axis	− 226.15 N
Total	2813 N

Minimum value over time	
X axis	452.56 N
Y axis	− 1475.7 N
Z axis	− 818.4 N
Total	651.84 N

From the results of the simulation analysis, it can be seen that the difference between the tangential force values of equal base circle herringbone bevel gears and Gleason is not significant, as the tangential force is mainly related to the torque applied at the main wheel and the distance from the gear meshing point to the main shaft center, under the same torque application. Due to the assembly, there is a deviation in the value. Therefore, the magnitude of the component force along the x-axis is not taken as a reference standard.

Thus, for measuring the equal base circle herringbone curved bevel gear's advantage over Gleason, only the magnitude of the force in the direction of the y-axis and z-axis is considered.

It can be seen that the radial force and axial force of Gleason are several times greater than those of the equal base circle herringbone curved bevel gear under the same loading conditions. The radial force on the equal base circle herringbone curved bevel gear and the dividing force on the axial force cancel each other out, resulting in a smaller combined force. The analysis agrees with the results using the arc tooth bevel gear calculation formula. The equal base circle herringbone curved bevel gear has a strong bearing capacity in the tooth direction compared with the Gleason, which improves its bearing capability. It can improve the phenomenon of broken teeth and increase the gear life in the axial direction.

## Conclusions

The design of an equal base circle herringbone curved bevel gear is based on the theory of equal base bevel gears. By establishing accurate 3D models, the assembly of the pinion and gear was validated in terms of the structure's feasibility and the principle's correctness. In this way, it provides an excellent theoretical analysis of gear contact axial forces. Compared with other bevel gears, it can be concluded that Equal-base circular herringbone bevel gear combines the advantages of herringbone and bevel gear. It is a bevel gear with a high overlap degree, strong bearing capacity, and long service life, which has advantages in gear transmission performance. A new tooth shape is proposed, and the article is geometrically designed with a small axial force as the design objective. On this basis, error sensitivity analysis, TCA, LTCA analysis, and dynamic characteristics of the gears after reshaping are required to improve the entire design and analysis process of this new gear and to lay the theoretical foundation for the practical application of this type of gear.

## Data Availability

The datasets generated during and/or analysed during the current study are available from the corresponding author on reasonable request.
